# Unfolded protein responses in T cell immunity

**DOI:** 10.3389/fimmu.2024.1515715

**Published:** 2025-01-08

**Authors:** Wencan Zhang, Xu Cao

**Affiliations:** ^1^ Shanghai Key Laboratory of Veterinary Biotechnology, School of Agriculture and Biology, Shanghai Jiao Tong University, Shanghai, China; ^2^ Shanghai Frontiers Science Center for Drug Target Identification and Delivery, and the Engineering Research Center of Cell and Therapeutic Antibody of the Ministry of Education, School of Pharmaceutical Sciences, Shanghai Jiao Tong University, Shanghai, China

**Keywords:** endoplasmic reticulum stress, unfolded protein response, T cell activation, T cell differentiation, immune regulation

## Abstract

Endoplasmic reticulum (ER) stress and the unfolded protein response (UPR) are integral to T cell biology, influencing immune responses and associated diseases. This review explores the interplay between the UPR and T cell immunity, highlighting the role of these cellular processes in T cell activation, differentiation, and function. The UPR, mediated by IRE1, PERK, and ATF6, is crucial for maintaining ER homeostasis and supporting T cell survival under stress. However, the precise mechanisms by which ER stress and the UPR regulate T cell-mediated immunity remain incompletely understood. Emerging evidence suggests that the UPR may be a potential therapeutic target for diseases characterized by T cell dysfunction, such as autoimmune disorders and cancer. Further research is needed to elucidate the complex interactions between ER stress, the UPR, and T cell immunity to develop novel therapeutic strategies for T cell-associated diseases.

## 1 Introduction

Endoplasmic Reticulum (ER) is a vital eukaryotic organelle that plays a key role in protein synthesis, folding, and post-translational modifications, as well as in lipid metabolism. If protein-folding burden is overwhelmed the ER’s capability, resulting in ER stress and an accumulation of misfolded proteins in its compartment, it initiates a cellular stress response termed the Unfolded Protein Response (UPR) (1). The UPR is a corrective mechanism aimed at mitigating the stress by reducing overall protein synthesis, boosting the ER’s folding capabilities, and degrading improperly folded proteins. Should the stress become unmanageable, the UPR may initiate cell death pathways (2, 3). During immune responses, the expansion and maturation of T cells require substantial protein production (4). It is thus not unexpected that accumulative evidence is highlighting the role of the UPR in the regulation of T cell destiny and related diseases. However, the underlying mechanisms and specific molecular targets involved remain insufficiently explored. In this review, we have delved into the effects of ER stress on the activation, differentiation, and function of T cells, with the goal to provide direction for future studies and therapeutic approaches to these conditions.

## 2 UPR: a process to determine cell live or die

**Figure 1:**
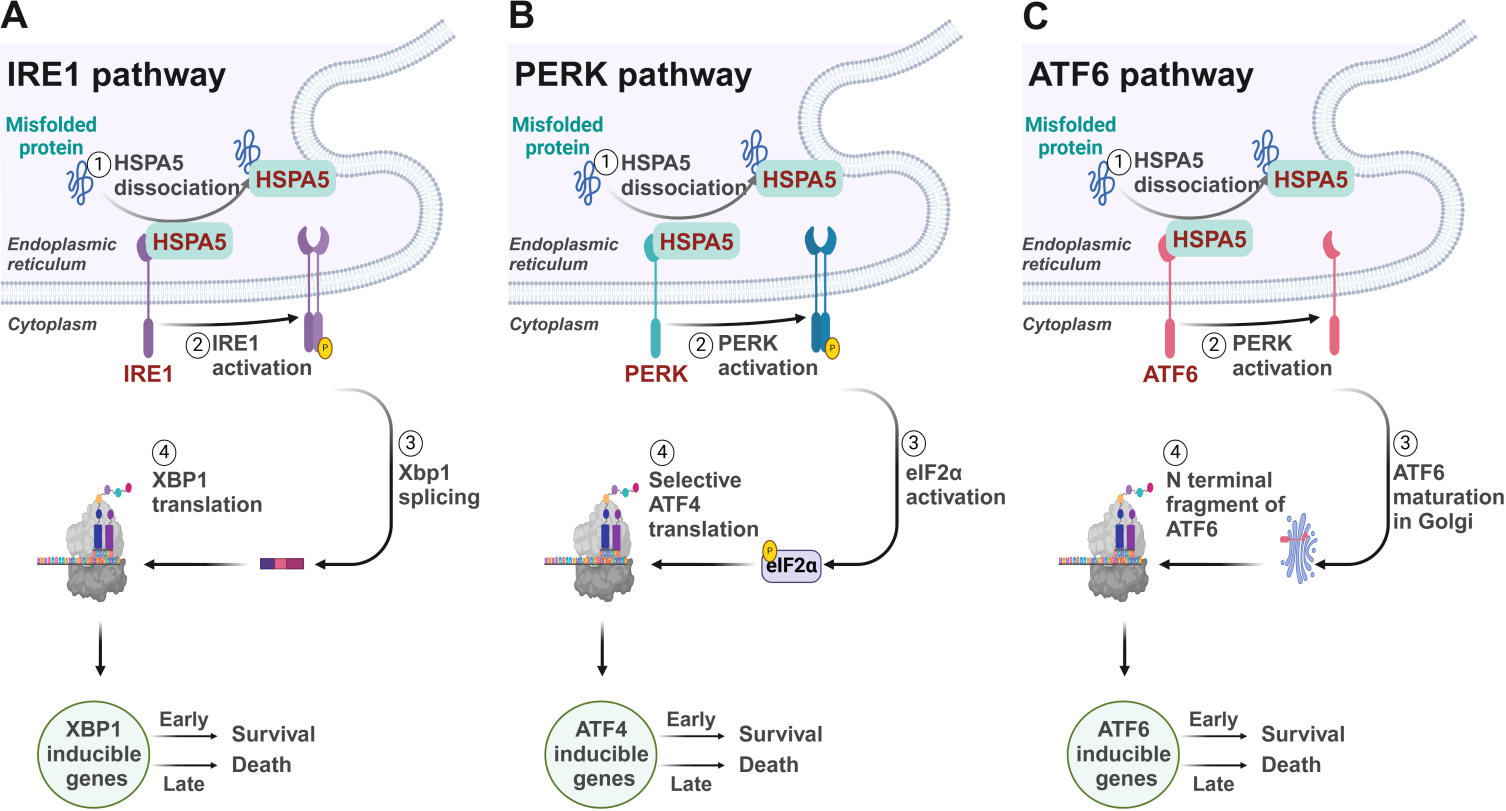
Signaling mechanisms of the unfolded protein response. **(A)** IRE1/XBP1 pathway. IRE1 initiates the splicing of XBP1 mRNA to produce the active transcription factor sXBP1, which upregulates genes crucial for protein folding and ERAD within the ER. **(B)** PERK/ATF4 pathway. PERK activates eIF2α phosphorylation, selectively boosting translation of ATF4. ATF4 mediates the downstream gene expression to reduce overall protein synthesis to ease ER folding stress. **(C)** ATF6 pathway. Under ER stress, ATF6 translocates to the Golgi where it is cleaved by S1P and S2P, releasing a domain that acts as a transcription factor in the nucleus, upregulating ER stress response genes to manage protein misfolding.

The UPR, the cellular reaction to ER stress, is a conserved mechanism across species designed to maintain ER homeostasis and ensure cell survival. This intricate process is activated to alleviate the ER’s protein-folding burden and to rebuild cellular balance (5). However, if the stress is not effectively managed, the UPR can initiate apoptotic pathways, underscoring its dual role in both sustaining and terminating cell life (**Figure 1**).

The UPR is regulated by three key proteins residing in the ER: inositol-requiring enzyme 1 (IRE1), PKR-like ER kinase (PERK), and activating transcription factor 6 (ATF6) (6). In the absence of stress, these proteins are maintained in an inactive form by the binding with the chaperone BiP. However, upon unfolded protein accumulates within the ER, it promotes the release of BiP, thereby activating these sentinels and initiating the UPR (7).

IRE1, a bifunctional kinase and RNase, plays a critical role in the UPR by initiating a signaling cascade that leads to the splicing of the X-box binding protein 1 (XBP1) mRNA. This splicing is a pivotal step in the UPR, as it generates a stable, spliced active transcription factor, sXBP1, which subsequently enhances the expression of genes that play a role in protein folding and the process of ER-associated degradation (ERAD) (8, 9). These genes include those encoding for glucose-regulated proteins (GRPs) and protein disulfide isomerases (PDIs) (10), which are crucial for managing the protein load within the ER.

Concordantly, the kinase PERK catalyzes the phosphorylation of eukaryotic initiation factor 2 alpha (eIF2α) on the serine 51 residue, which diminishes global protein synthesis by limiting the formation of the eIF2α-GTP-tRNAi complex required for translation initiation (11). This reduction in protein synthesis alleviates the ER’s folding demand and provides temporary relief. Meanwhile, PERK selectively enhances the translation of upstream open reading frames (uORFs) containing mRNAs (12), such as those for the transcription factor ATF4 (13). ATF4 then activates an array of genes that participate in the metabolism of amino acids and the production of antioxidant proteins (14–16), which are essential for resolving ER stress.

ATF6 is also activated under ER stress conditions leading to its translocation to the Golgi apparatus (17). There, ATF6 undergoes proteolytic cleavage by site-1 protease (S1P) and site-2 protease (S2P), releasing the cytosolic domain of ATF6 (18). This domain then relocates into the nucleus to function as a transcription factor, driving the upregulation of genes involved in the ER stress response, including those for chaperones like GRP78 and GRP94, and components of the ERAD machinery (19–21). ERAD ensures the efficient degradation of misfolded proteins, thus preventing their accumulation within the ER (22, 23).

The intricate interplay among IRE1, PERK, and ATF6 involves multiple layers of regulation, allowing these proteins to respond to immediate cellular demands during ER stress and to modulate long-term adaptations that enable the cell to manage chronic stress. However, if ER stress is excessive or persistent, these adaptive responses may fail, leading to cell death through apoptosis (24). The decision to initiate apoptosis is influenced by the balance between pro-survival and pro-death signals, often involving the activation of the transcription factor CHOP (C/EBP homologous protein) and the induction of death receptor signaling (25). This balance is therefore critical in determining cell fate under conditions of ER stress.

## 3 The active role of UPR molecules in T cell biology

**Figure 2:**
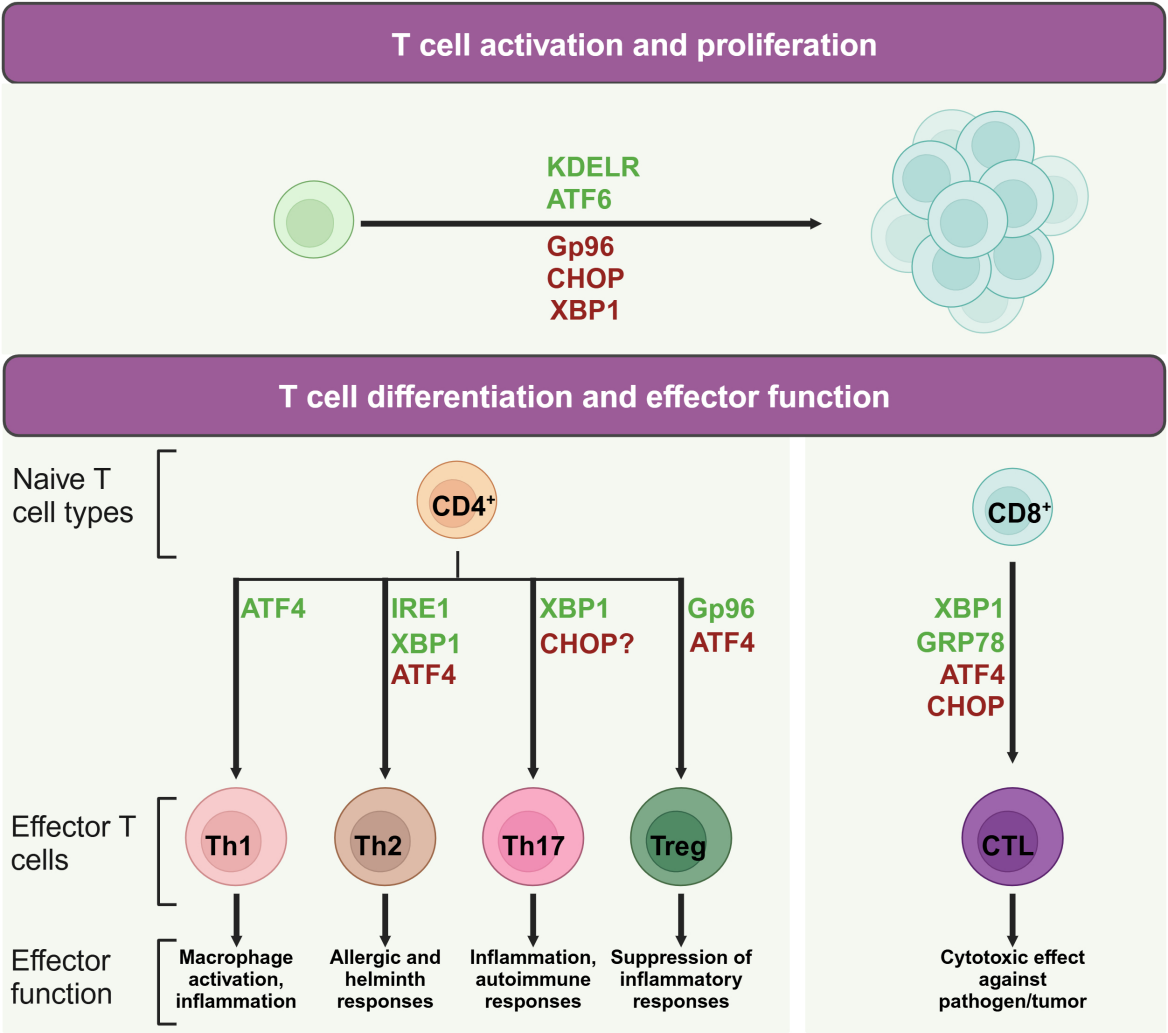
Role of UPR molecules in T cell activation and differentiation. During T cell activation and differentiation, UPR associated molecules play active roles in the life of T cells. Proteins in green may promote indicated process, while protein in red may suppress its activation or differentiation.

When naïve T cells contact with antigen-presenting cells (APCs) carrying appropriate peptide epitopes, they initiate a cascade of signals leading to their activation, replication, and differentiation (26, 27). Given the critical role of protein synthesis in T cell activation, UPR are increasingly recognized as key players in regulating T cell function and immunity. It not only supports T cell survival and function by maintaining ER homeostasis but also has inhibitory effects. For example, certain UPR molecules, such as CHOP, can reduce naïve T cell numbers and influence their quiescence (28), while IRE1 promotes CD4^+^ T cell activation (29). This underscores the need for a comprehensive summary of UPR-related molecules in T cells immunity (**Figure 2**).

### 3.1 UPR molecules and T cell activation

When T cells contact with an APC, the T cell receptor (TCR) activation induces a series of intracellular pathways that result in the activation of the UPR. This response is designed to rebuild ER homeostasis by upregulating the expression of chaperone proteins, enhancing protein folding capacity, and promoting the degradation of unfolded or misfolded proteins (30).

Upon the activation of TCR signaling, ER stress and the UPR are integral to the balance and expansion of T cell populations. For instance, the ER chaperone protein Gp96, which also functions as a calcium buffering protein, is upregulated in response to TCR stimulation (31). CD4^+^ T cell specific deletion of Gp96 has been found to impair glycolytic ability due to defective calcium mobilization upon TCR engagement (31), highlighting the importance of ER-modulated cytosolic calcium in the early expansion of naïve CD4^+^ T cells. Moreover, the KDEL receptor 1 (KDELR) is tasked with the return of ER-localized proteins to the ER from the cis-Golgi network (32). Research on cell models overexpressing a defective KDELR has illuminated how the failure to restore ER proteins can lead to an increased UPR (33). Murine T cells with a genetic alteration in KDELR display heightened eIF2 phosphorylation and CHOP expression, which correlates with a decrease in naïve T cell counts *in vivo* (28). However, A robust TCR activation can counteract the deficiencies caused by KDELR mutations, thereby preserving the population of immature T cells and their capacity for proliferation (28).

ATF4, which is activated by eIF2 phosphorylation, is also implicated in driving T cell proliferation. Mice with a deficiency in ATF4 in their T cells exhibit reduced proliferation rates (15). Meanwhile, XBP1 and CHOP are implicated in maintaining T cell dormancy (34). In mice with a mutation in Schlafen-2 (Slfn2), a higher ratio of T cell mortality has been observed due to chronic ER stress (35, 36). The removal of XBP1 or CHOP in these mice enhances their survival rates and partially rectifies developmental irregularities (36), underscoring the intricate dynamics between ER stress and T cell expansion.

### 3.2 UPR molecules and CD4^+^ T helper cells

CD4^+^ T cells play central roles in human immunity. Throughout the process of clonal expansion, a potent CD4^+^ T cell-driven immune reaction is contingent upon the formation of various specialized subsets, including Th1, Th2, Th17, and regulatory T cells (Tregs) (37). In recent studies, the UPR and ER stress are recognized as pivotal regulators that shape the maturation and functional dynamics of T helper lymphocytes.

Th1 cells notably produce proinflammatory cytokines such as interferon-gamma (IFN-γ) and tumor necrosis factor-alpha (TNF-α) (38). Studies have shown that IFN levels are significantly lowered in splenocytes from ATF4-deficient mice subjected to autoimmune disease models (15, 39). The most prominent ER stress regulators involved in Th1 differentiation is ATF4. ATF4, while not essential for thymic T cell maturation, exhibits critical regulatory roles in the functional execution of Th1 cells, as evidenced by research (15, 40). Utilizing a murine model involving the transplantation of fetal liver cells, Yang et al. revealed that the deletion of ATF4 culminates in compromised Th1 functionality across varying oxidative conditions (15). The activation of ATF4 in T cells, especially under high oxidative stress or amino acid limitation, is partially driven by GCN2 (15), which initiates the UPR by phosphorylating eIF2 in response to cellular stressors like proteasome inhibition and nutrient scarcity (41). Furthermore, ATF4 influences the mTORC1 pathway, with its inhibition leading to a reduction in mTORC1 signaling (15), a known promoter of Th1 cell differentiation (42). Conversely, IRE1 and PERK do not appear to directly influence Th1 functionality, as their genetic or pharmacological suppression does not impede the manifestation of Th1 effector capabilities (29, 43). This observation implies that ATF4 may be activated through alternative eIF2 phosphorylation-inducing pathways, not solely dependent on PERK for its upregulation in Th1 cells.

Upon stimulation with IL-4, naive CD4^+^ T cells are differentiated into Th2 cells. These cells are pivotal in humoral immunity, secreting cytokines such as IL-4, IL-5, IL-9, and IL-13 (44). The signaling pathway mediated by IRE1α-XBP1 is associated with increased release of cytokines IL-4, IL-13, and IL-5 in Th2 cells, suggesting a role for this pathway in modulating the Th2 cell secretory profile (45, 46). However, compound 4u8c, an inhibitor of IRE1α-XBP1 pathway, inhibits IL-4 production in maturing CD4^+^ T cells without affecting established Th2 cells (47). Moreover, the inhibition of the IRE1α-XBP1 pathway can arrest Th2 cells in the phase of S or G2/M (29), indicating its role in promoting Th2 differentiation by reducing protein secretion demands and accelerating cell proliferation. In addition, following TCR activation, there is a notable increase in the phosphorylation of eIF2α and the upregulation of ATF4 in Th2 cells, which in turn affects the expression of downstream genes such as GADD34, CHOP, GRP78, and HERP (48). Th2 cell re-stimulation can result in the swift eIF2α dephosphorylation, thereby triggering the synthesis of IL-4 (48).

Th17 cells are defined by their unique cytokine profile, prominently secreting IL-17A, IL-17F, along with IL-22. These cells exert significant influence on inflammatory responses and the pathogenesis of autoimmune conditions (49). Evidence suggests a link between CHOP and the control of IL-17 expression. Yet, contemporary research has noted that mice lacking CHOP display unaltered Th17 cell maturation (50, 51). Studies discovered that physiological stress induced by hypoxia, ionic tension changes, and glucose scarcity can stimulate XBP1 to initiate the UPR, thereby promoting the formation of Th17 cells (52, 53). Conversely, cellular stress alleviation with Tauroursodeoxycholic acid (TUDCA), a bile acid that mitigates ER stress, has been observed to slow the progression in a model of multiple sclerosis, indicating the pivotal role of ER stress in Th17 cell differentiation (54). Furthermore, the study indicated that compounds triggering ER stress can intensify Th17 effector functions (54). Notably, the study also revealed a delayed disease onset in mice with lymphocytes lacking XBP1 (54), aligning with findings that highlight the induction of Th17 effector functions under hypoxic and osmotic stress conditions (52, 53).

ER stress plays a significant role in Treg cells which are pivotal in immunosuppression (55). Abolishing the chaperone gp96 leads to a decrease in Foxp3 levels within Treg cells, along with a diminished output of active TGF-β from its latent form, latent membrane-associated TGF-β (56). Consequently, the absence of gp96 induces Treg lineage instability and diminishes the *in vivo* suppressive functions. In parallel, Treg cells deficient in Hrd1 exhibit heightened IRE1α levels, triggering the MAPK p38 cascade, which hinders Foxp3 synthesis and thus undermines Treg stability (57). When encountering severe ER stress, Treg cells are capable of increasing the expression of Foxp3, IL-10, and TGF-β, facilitated by the PERK signaling (43). Mouse CD4^+^ T cells lacking ATF4 has shown increased Foxp3 transcript levels especially under conditions promoting Treg cell microenvironment and characterized by oxidative stress (15).

### 3.3 UPR molecules and CD8^+^ cytotoxic T cells

CD8^+^ T lymphocytes, integral to immune surveillance against cancer and pathogens (58), undergo differentiation processes influenced by ER stress. This stress response is instrumental in shaping the functionality of CD8^+^ T cells. Experiments with Lymphocytic choriomeningitis virus-infected mice demonstrated increased levels of the spliced form of XBP1, which fostered the maturation of CD8^+^ T cells (59). The upregulation of CHOP, a consequence of the PERK-ATF4 signaling cascade, results in the impairment of CD8^+^ tumor-infiltrating lymphocytes through the suppression of T-bet expression (60). Furthermore, the ER stress-associated chaperone GRP78 has been shown to regulate granzyme B activity of cytotoxic T cells (CTL) in their intraepithelial counterparts (61). Deletion of GRP78 from CTL also displayed lowered granzyme B secretion and compromised cytotoxic potential. This reduction was linked to attenuated proliferation driven by IL-2, but the supplementation with exogenous IL-2 partially ameliorated the decrease in granzyme B levels (62), underscoring the complex interplay between ER stress and cytotoxic T cell function.

## 4 UPR in T cell-associated autoimmune disease and cancer

Given the crucial function of the UPR in T cell activation and differentiation, a growing body of evidence indicates that UPR plays a significant role in the development of T cell-related diseases. This paragraph synthesizes the current understanding of UPR’s involvement in autoimmune diseases and cancer.

### 4.1 Autoimmune diseases

In systemic lupus erythematosus (SLE), while the disease is often manifested by the production of autoantibodies against nuclear antigens and self-proteins, it is the dysfunction of T cell activity and the imbalance of T cell subsets that significantly contribute to the condition (63). Additionally, the disease process is partly driven by increased rates of T cell apoptosis (64). As the UPR is crucial for the efficacy of T cell functions, as previously discussed, strategies targeting UPR-related molecules in T cells might offer protective effects against SLE. T cells derived from individuals with SLE exhibit modifications in molecular adhesion patterns, intracellular signaling mechanisms, and components of the TCR complex (65). SLE perturbs the equilibrium of T cells, a process where the UPR exerts regulatory influence. Studies indicate that oxidative stress might contribute to T cell impairments in SLE, activating UPR-associated genes in response to this stress (66). Decreased levels of CHOP, IRE1, and PERK in peripheral blood mononuclear cells (PBMCs) of SLE patients have been reported, contrasted with the upregulation of total XBP1 and its spliced form (67). Given that T cells account for 50–75% of PBMCs in healthy individuals, the elevated gene expression observed may be partially attributed to the presence of T cells in individuals with SLE (67). Moreover, T cells in individuals with SLE are more susceptible to apoptosis triggered by ER stress (64), providing a possible strategy to target ER stress in T cell for SLE therapy.

In rheumatoid arthritis (RA), antibodies to GRP78, GRP94, and Calnexin are found in patient sera (68). In particular, alongside the upregulation of GRP78 in RA patients’ synovial fluid, reactive T cells to this molecule are also detected (69). Moreover, both preimmunization murine subjects with GRP78 protein (70) and administration of GRP78 molecules during arthritis progression (71) significantly mitigated the severity of disease along with upsurge of IL-4, IL-5, and IL-10, suggesting a causal role of GPR78 in RA pathology. Moreover, elevated activity within the IRE1/XBP1 signaling network has been observed in fibroblasts from rheumatoid arthritis patients, with IRE1’s role being instrumental in the disease by maintaining the stability of cytokine mRNA transcripts (72). Moreover, the heightened expression of GADD34, a consequence of the PERK/eIF2α/ATF4 signaling route, is linked to the excessive production of pro-inflammatory cytokines in the context of RA (73). In addition, ER stress associated autophagy is suggested to alter the characteristics of fibroblasts in RA, where the IRE1/JNK signaling axis is crucial for boosting cellular division, movement, and the ability to infiltrate surrounding tissues (74). These together indicates that targeting UPR might stand as a viable approach for the therapy of RA.

In individuals with type 1 diabetes, the depletion of pancreatic β cells is intricately linked to the density of CD8^+^ T cells that have infiltrated the pancreatic islets (75). As the islets experience escalating T cell infiltration, there is a substantial release of IL-1β and TNF-α, triggering endoplasmic reticulum (ER) stress in β cells (75). When the capacity for UPR-mediated compensation is overwhelmed, it triggers apoptosis in β cells. Under ER stress, IRE1α in β cells undergoes activation, forming homodimers, autophosphorylation, and subsequent phosphorylation by TRAF2, ASK1, and JNK (76). The activation of JNK initiates apoptosis in β cells through various signaling cascades. The expression of CHOP is heightened through the IRE1α/JNK/CHOP signaling axis, contributing to β-cell demise (77). Furthermore, IRE1α can directly activate caspase-12, leading to β cell death in a rat model of virus-induced diabetes (78). Additionally, IRE1α engages NF-κB by interacting with TRAF2, which in turn upregulates the production of multiple cytokines and chemokines, facilitating β cell apoptosis (79). In the PERK pathway, the activation of PERK leads to the phosphorylation of eIF2α, prompting ATF4 transcription and translation. ATF4, in turn, enhances the expression of ATF3 and CHOP, both of which are implicated in β cell apoptosis (80). Overstimulation of ATF6 results in the suppression of insulin gene expression, culminating in β cell dysfunction and death (81). The inflammatory cytokines IL-1β and IFN-γ impede mTOR and activate the AMPK-ULK-1 pathway to initiate autophagy (82). However, these same cytokines also reduce lysosomal function, thereby inhibiting autophagy and intensifying ER stress, which ultimately leads to β cell apoptosis (82).

### 4.2 Cancer

Despite of the crucial role in combating cancer, T cells within the tumor microenvironment (TME) often confront restrictive metabolic conditions that may impair their capacity to effectively execute their tumor-killing functions (83, 84). Due to shortage in glucose in TME, tumor infiltrating lymphocytes (TILs) often experience ER stress suppressing protein translation. Research has shown that proteasome activators can boost the antitumor immunity of TILs through mitigating the activation of PERK-p-eIF2α (85). Likewise, XBP1 is often found to be upregulated in TILs, thereby reducing the abundance of TILs and also a decrease of IFN-γ level (86). As a result, T cell-specific XBP1 knockout mice have shown resistance to engraftment of ovarian cancer due to strong CD8^+^ T cell mediated antitumor immunity (86). Moreover, increased CHOP levels were identified within CD8^+^ TILs in ovarian cancer, which was linked to the activation of the PERK signaling axis and the promotion of ATF4, consequently suppressing T-bet expression (60). In the context of pancreatic cancer, T cells interact with exosomes released by tumor, leading to the activation of the p38 MAPK pathway, thereby initiating ER stress. This event sets off a cascade that activates the PERK-eIF2α-ATF4-CHOP signaling cascade, culminating in the induction of T cell death (87). In liver cancer, the limitation of glutamine in the TME results in diminished GLS2 expression within CD8^+^ T cells, consequently triggering ER stress and impairing the cytotoxic T cells’ capacity to eliminate cancer cells (88).

## 5 Conclusions

The intricate relationship between ER stress and T cell functions is a burgeoning area of research with significant implications for immunology. As T cells undergo developmental and activation processes, the ER’s protein-folding capacity can become overwhelmed, triggering ER stress and activating the UPR. We have underscored the UPR’s role in sustaining T cell homeostasis and its potential influence on diseases that involve T cell mediation. While the current body of research has shed light on the importance of ER stress in T cell biology, there is a recognized gap in understanding the detailed mechanisms by which the UPR influences T cell immunity. The UPR’s influence on T cell development and its potential as a therapeutic target in T cell-driven pathologies warrants further exploration (30, 89). More studies should aim to dissect the complex interplay between the UPR, T cell differentiation, and immune function, which could unveil new avenues for intervention in diseases characterized by aberrant T cell activity. This pursuit of knowledge may lead to innovative strategies that harness the UPR’s regulatory capabilities, offering novel therapeutic approaches to modulate T cell responses and treat associated immunological disorders.
